# Antiplatelet effects of citalopram in patients with ischaemic stroke: A randomized, placebo-controlled, double-blind study

**DOI:** 10.1038/s41598-019-56487-8

**Published:** 2019-12-27

**Authors:** Kristian Lundsgaard Kraglund, Janne Kaergaard Mortensen, Søren Paaske Johnsen, Grethe Andersen, Erik Lerkevang Grove

**Affiliations:** 1 Department of Neurology, Aarhus University Hospital, Aarhus, Denmark; 2 Department of Neurology, Vestfold Hospital, Tønsberg, Norway; 3 Danish Center for Clinical Health Services Research, Department of Clinical Medicine, Aalborg University, Aalborg, Denmark; 4 Department of Cardiology, Aarhus University Hospital, Aarhus, Denmark; 5 Department of Clinical Medicine, Faculty of Health, Aarhus University, Aarhus, Denmark

**Keywords:** Stroke, Outcomes research, Neurology

## Abstract

We evaluated the effect of SSRI treatment on platelet aggregation in patients with ischaemic stroke and included patients from the randomized double-blind controlled study of citalopram in acute ischaemic stroke (TALOS). Patients on clopidogrel were included 6 months after acute ischaemic stroke. Platelet parameters, including P2Y12 platelet reactivity using the VerifyNow System, were measured at the last day of study treatment and repeated after a 14-day wash-out period. A total of 60 patients were included (n = 32 randomized to citalopram). Platelet aggregation levels did not differ between the citalopram group (mean 116, 95% CI 89 to 143) and the placebo group (mean 136, 95% CI 109 to 163) (On-treatment, p = 0.14). Similarly, there was no significant change in platelet aggregation in the citalopram group from on-treatment to post-treatment (mean difference 2.0; 95% CI −18 to 14). Platelet count, size and turnover were not affected by SSRI treatment. In conclusion, SSRI therapy did not lead to statistically significant inhibition of platelet aggregation in ischaemic stroke patients treated with clopidogrel.

## Introduction

Selective serotonin reuptake inhibitors (SSRIs) are widely used after stroke^1,2^. Observational studies have indicated an increased bleeding risk and a protective effect of SSRIs against recurrent cardiovascular events in patients with ischaemic stroke.

Platelets lack the enzyme needed to produce serotonin (5-HT), but a 5-HT transporter (5-HTT) enables rapid uptake from plasma^3^. If a thrombotic event occurs, 5-HT is released^3^. 5-HT is a relatively weak platelet activator *per se*, but facilitates platelet response to other agonists, including adenosine diphosphate (ADP)^4^. SSRIs inhibit the 5-HTT and reduce platelet 5-HT concentrations by almost 90%. This inhibits platelet-mediated haemostasis and may reduce the risk of cardiovascular events^5,6^.

This is a predefined substudy (Ethical committee approval no 1-10-72-183-13, see also supplementum in^7^) of the TALOS trial testing the SSRI citalopram in non-depressed patients with acute ischaemic stroke^7,8^. TALOS showed similar risks of cardiovascular events between treatment groups and a borderline statistical significant effect on functional recovery in the on-treatment analysis. Clopidogrel is frequently used as secondary prevention in patients with ischaemic stroke^9,10^. We compared platelet aggregation levels in patients with ischaemic stroke treated with clopidogrel in addition to citalopram vs placebo. We hypothesized that patients treated with citalopram would have lower platelet aggregation levels compared to placebo-treated patients. Also, that platelet aggregation would increase in patients randomized to citalopram when measurements were repeated after a wash-out period.

## Materials and Methods

### Study design and subjects

Patients were included from the TALOS trial: an investigator-initiated randomized, controlled, double-blind trial^7,8^. In brief, patients with first-ever ischaemic stroke within the previous 7 days were enrolled from three Danish stroke units. All patients were treated in accordance with stroke guidelines and were randomized to either citalopram or placebo. A total of 60 consecutive patients treated with clopidogrel were included 6 months after acute ischaemic stroke as described below.

The study was investigator-driven and conducted according to the principles of the Declaration of Helsinki and monitored according to Good Clinical Practice guidelines. Written informed consent was obtained from all participants. The protocol was approved by the Committees on Health Research Ethics (1-10-72-183-13), EudraCT number is 2013-002253-30 and clinical trial registration number (clinicaltrials.gov) for the main TALOS study is NCT01937182.

### Study medications

Patients were randomly assigned 1:1 to receive either citalopram (*Sandoz*, Copenhagen, Denmark) administered orally once daily for 6 months at a dose of 20 mg (10 mg if aged ≥65 years and/or reduced liver/kidney function) or placebo. In order to standardize the time between drug ingestion and platelet function testing, intake of the study drug and clopidogrel (75 mg once daily) was scheduled to occur at 8 a.m. on the day before and at the day of testing.

### Blood sampling

Patients were scheduled for follow-up between noon and 3 p.m. on the last day of treatment with study drug (*On-treatment*) and 14 days later (*Post-treatment*). Using minimal stasis, blood samples were taken from a forearm vein using a 21-gauge needle:

1. 6 mL tube (discharged to avoid stasis-induced platelet activation).
2. Vacuette 9NC Coagulation Sodium Citrate 3.2% 2 mL tube (VerifyNow testing).
3. Vacutainer 6 mL (centrifuged for 15 min at 1000 × g at 5 °C and stored at −80 °C for later analysis).
4. Vacutainer tube K2EDTA 10 mL (other analyses including platelet count and reticulated platelets).

### Platelet aggregation measurements

Platelet aggregation was measured using the VerifyNow P2Y_12_ platelet reactivity system (*Accriva Diagnostics*, San Diego, CA, USA). VerifyNow is a turbidimetric-based optical detection system measuring platelet aggregation in whole blood^11^. The VerifyNow has high reproducibility^12^. Results are reported as P2Y12 Reaction Units (*PRU*).

### Other platelet parameters

Using a Sysmex XN-8000 (*Sysmex*, Kobe, Japan), we measured total platelet count, immature platelet count, platelet-large cell ratio, mean platelet volume, white and red blood cell count, haemoglobin, and reticulocytes.

### Statistical analyses

Samples from the two groups (citalopram vs placebo) were compared using student’s t-test. P-values below 5% were considered statistically significant. No previous studies in similar populations were available before study initiation and, therefore, no formal sample size calculation was performed. Based on previous platelet function studies using the VerifyNow, a total of 25–30 patients in each group was considered appropriate, and consecutive patients were asked to participate, until 60 patients had been included. Statistical analyses were performed using Stata 13.1 (*StataCorp*, College Station, TX, USA).

## Results

The two groups were well balanced with respect to age ((mean (SD), 71 (9.3) years), 38% females). The median time from intake of study medication to blood sampling did not differ between groups: 5.1 h in the citalopram group and 4.8 h in the placebo group.

ADP-induced platelet aggregation (PRU) levels did not differ between the citalopram group (mean 116, 95% CI 89 to 143) and the placebo group (mean 136, 95% CI 109 to 163, p = 0.14) when comparing patients during treatment (Table 1). *On-treatment* PRU values in the citalopram group (mean 116, 95% CI 89 to 143) did not differ from the values measured *Post-treatment* (mean 114; 95% CI 88 to 139). Other platelet parameters are presented in Table 1. Platelet turnover, quantified using immature, reticulated platelets^13^, showed no significant difference between groups or between measurements in the citalopram group. Haemoglobin levels were significantly lower in patients allocated to citalopram treatment. This was observed both for the *On-treatment* (p = 0.041) and the *Post-treatment* measurement (p = 0.001).

**Table 1:** Haematology parameters measured during treatment with both project medication and clopidogrel (On-treatment) and during treatment with clopidogrel monotherapy (=Post-treatment).

Test, units	On-treatment			Post-treatment		
	Placebo *n* = 28	Citalopram *n* = 32	p value	Placebo *n* = 27	Citalopram *n* = 32	p value
Platelets, 10^9^/L	241	271	0.89	240	281	0.97
Immature platelet count, 10^9^/L	7.1	8.1	0.80	6.5	7.7	0.90
Immature platelet fraction, %	3.0	3.0	0.12	2.9	2.8	0.46
White blood cell count, 10^9^/L	6.9	6.9	0.44	7.2	6.9	0.31
Red blood cell count, 10^12^/L	4.7	4.6	0.10	4.7	4.5	0.014
Haemoglobin, mmol/L	9.0	8.6	0.041	9.0	8.3	0.001
Mean platelet volume, fL	10.3	10.2	0.30	16.2	10.2	0.14
Platelet-large cell ratio, %	0.27	0.26	0.28	0.3	0.3	0.28
Reticulocytes, 10^9^/L	50.0	53.1	0.78	51.7	48.9	0.25

Calculations are based on mean values. fL: femtolitre (10^−15^ litres).

## Discussion

In this predefined substudy of the randomized controlled trial TALOS, we evaluated potential antiplatelet effects of the SSRI citalopram in ischaemic stroke patients treated with the P2Y_12_ inhibitor clopidogrel for prevention of recurrent stroke. The primary outcome, PRU, was measured during treatment with clopidogrel and citalopram/placebo and again after wash-out. Platelet function testing was performed after a standardised time interval between drug ingestion and blood sampling, which was performed at the same time of the day to minimise any diurnal variation in platelet aggregation. Platelet function testing did not show any significant difference in PRU between groups at both measurements (i.e. during combined therapy and on clopidogrel monotherapy after a wash-out period). Similarly, no difference was observed between patients allocated to citalopram, when measurements were taken on *on-treatment* and repeated *post-treatment*.

Previous studies have indicated an increased risk of bleeding, when SSRIs are given in combination with aspirin and/or clopidogrel^14^, but these observational studies are to some extent hampered by confounding-by-indication. This is the first randomized study investigating platelet aggregation levels in patients receiving citalopram/placebo during concomitant treatment with clopidogrel, and the study thus provides novel information with respect to the cardiovascular benefits and risks with SSRI. The design of the TALOS study allowed repetitive measurements in the same patients receiving either citalopram or placebo in combination with clopidogrel, providing a unique opportunity to analyse citalopram’s platelet inhibitory effect.

*In vitro* studies have reported 5-HT as a weak platelet agonist *per se*^15^, and 5-HT primes platelet activation and potentiates procoagulant responses of platelets, including that from ADP^16^. In addition, studies have shown decreased platelet aggregation in 5-HTT knockout mice^17^.

The standardisation of blood sampling kept the variability from other parameters to a minimum. Importantly, all study subjects were treated with clopidogrel, which may have reduced the ability to detect minor platelet inhibition by SSRIs. However, studying stroke patients without any antithrombotic treatment would entail ethical challenges. Moreover, platelet function testing was performed 6 months after ischaemic stroke, and one might hypothesize that platelet reactivity, and thus the potential for detecting inter-group differences when adding citalopram, would have been larger in the early phase after ischaemic stroke. In this population of stroke patients treated with clopidogrel mono antithrombotic therapy, we evaluated platelet function based on the ADP pathway, and it cannot be excluded that SSRIs may also inhibit platelet aggregation by non-ADP pathways. Accordingly, recent data indicate that citalopram reduce collagen-induced platelet aggregation by concentration-dependent inhibition of convulxin-related aggregation, serotonin release and activation of αIIbβ3^18^. Another recent study^19^ reported evidence for two novel mechanisms of citalopram-induced platelet inhibition, including inhibition of so-called CalDAG-GEFI/Rap1 signalling and competitive antagonism of glycoprotein VI in platelets. Finally, platelet aggregation levels were numerically lower during treatment with citalopram in our study, and results might have differed in a larger study population. The results of this platelet function study do not indicate that treatment with citalopram is beneficial or harmful in stroke patients treated with clopidogrel, but more studies are warranted to evaluate the effect of SSRIs on non-ADP dependent platelet function and to evaluate clinical implications of combined treatment with SSRIs and antithrombotic therapy.

In conclusion, although SSRI treatment may reduce the risk of recurrent cardiovascular events according to prior studies, our data do not support the hypothesis that citalopram provides substantial platelet inhibition in patients with ischaemic stroke treated with clopidogrel.

### Compliance with ethical standards

The Danish Council for Independent Research, Danish Regions Medical Research Council, and Central Denmark Region Research Council provided funding for this study, but had no role in the study design; in the collection, analysis and interpretation of data; in the writing of the article; or in the decision to submit the paper for publication.

Written informed consent was obtained from all participants. The study was conducted in accordance with the ethical standards of the national research committee and with the 1964 Helsinki declaration and its later amendments. Approvals were obtained from regulatory agencies and the Committees on Health Research Ethics as described above in the section on Methods.

## Data Availability

The datasets analysed for this study are available from the corresponding author upon reasonable request.
